# Discovery of dynamic tumor microenvironment architecture with targeted agents in the multiplex implantable microdevice assay

**DOI:** 10.1038/s41392-026-02824-z

**Published:** 2026-07-10

**Authors:** Juraj Jakubik, Zuzana Tatarova

**Affiliations:** 1 German Cancer Consortium (DKTK), partner site Frankfurt/Mainz, a partnership between DKFZ and Institute for Tumor Biology and Experimental Therapy, Frankfurt am Main, Germany; 2 German Cancer Research Center (DKFZ), Heidelberg, Germany

**Keywords:** Cancer microenvironment, Assay systems, Predictive medicine, Breast cancer, Cancer therapy

**Dear Editor**,

Progress in anatomical tissue imaging, alongside the development of advanced unsupervised learning algorithms for computational analysis, has unlocked substantial potential for enhanced characterization of tissue microstructure.^1,2^ While data-driven identification of cell clusters reduces subjective bias, the discovery of microanatomical domains often pertains to normal organs with inherent organization. Defining topological principles in tissues without strong structural patterning, such as cancer, has been limited, and deeper stratification in terms of identity and biological function, especially with treatment response, is challenging. Direct evidence and a side-by-side measure of therapy-induced changes could enable differential analysis development for patient stratification and personalized cancer medicine.

Recently, we introduced a Multiplex Implantable Microdevice Assay (MIMA^3^; Fig. 1a, left) that allows predicting effective combination treatments of targeted anticancer agents with immunotherapies. The system integrates a miniaturized drug-delivery device for localized intratumoral delivery of multiple treatments with computational analyses of multiplex imaging data for quantitative spatial evaluation of cancer, stroma, and different immune cells, including distinct macrophages, dendritic cells, neutrophils, and T cells in proximity to each treatment delivery site.^3^ We previously used supervised dimensionality reduction in hierarchical clustering to classify standard cell types and states of the complex tumor microenvironment (TME).^3^ With a specific focus on functionally broad targeted anticancer agents—panobinostat (pan-histone deacetylase inhibitor), venetoclax (BCL-2 inhibitor), and palbociclib (CDK4/6 inhibitor; Fig. 1a, right)—here we systematically investigate cell phenotypes and local neighborhoods using unsupervised architecture methods within MIMA to understand and identify topological TME principles in cancer therapy response (Fig. 1 and the online Repository Supplement; 10.6084/m9.figshare.32513355).

**Fig. 1:**
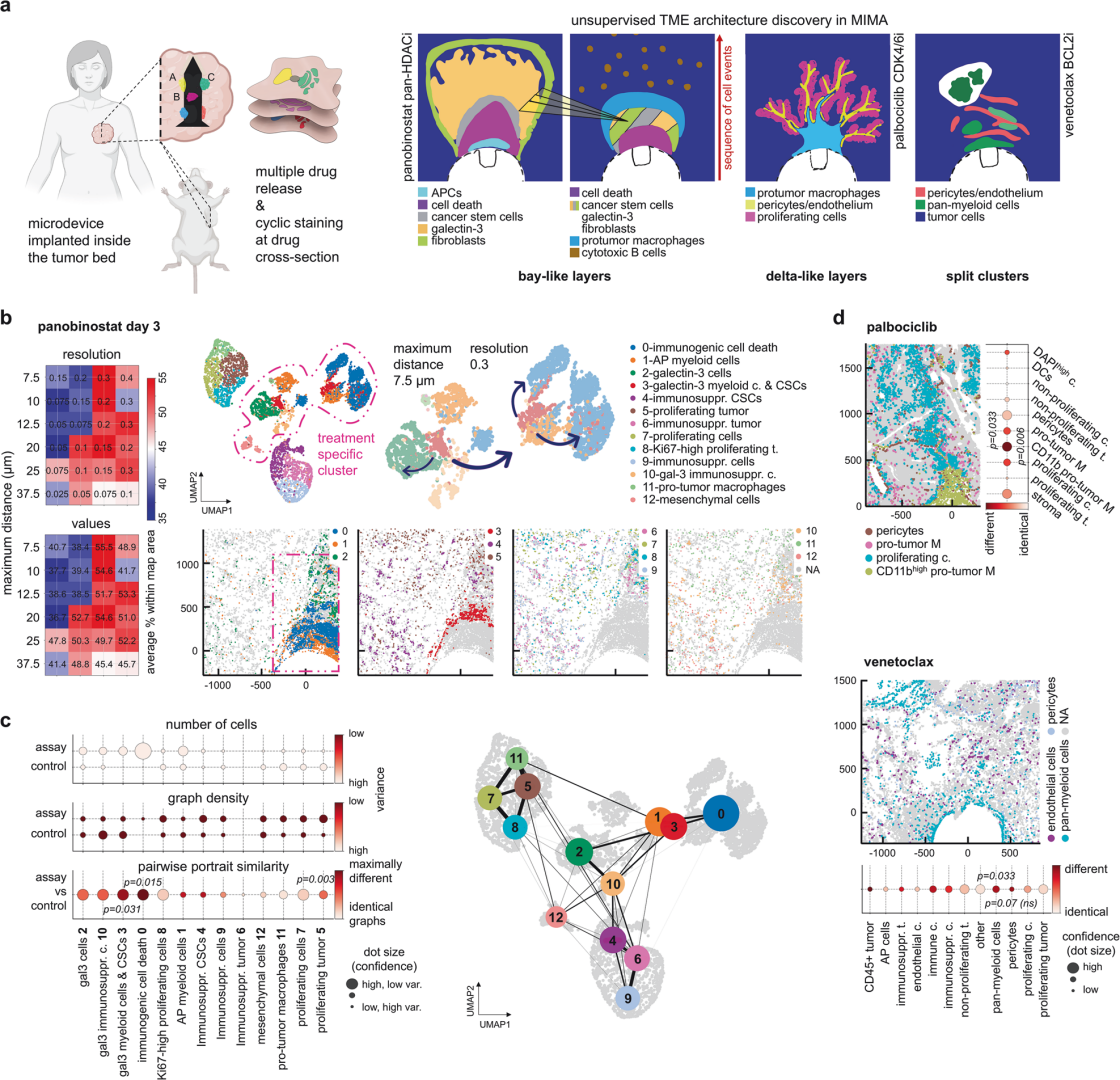
Integrated workflow for the multiplex implantable microdevice assay (MIMA) with spatial unsupervised learning to find treatment-induced tumor microenvironment anatomy. **a** *Left* MIMA: An implantable microdevice loaded with multiple drugs is gently implanted into the tumor bed through a biopsy needle or through a small incision in the skin in cancer patients and mice, respectively (schematic was partly generated with Biorender). The drugs are passively released into spatially separated regions, and the resulting formalin-fixed paraffin-embedded (FFPE) tissues are cut at the drug-tumor interface perpendicular to the device. Multiplex immunohistochemical staining is performed by cyclic staining, whole slide scanning and antibody stripping using a single FFPE slide for protein readout.^3^ *Right* Macroscopic schematic presentation of spatial patterns formed with localized panobinostat, palbociclib and venetoclax drug delivery. A cross-section is presented; the microdevice border is marked by a dashed line, and the drug is released from the microwells upward. Panobinostat induces bay-like layers with some phenotypes disappearing, merging, and appearing over time. Shared phenotypes across time served as an anchor providing insights into the sequence of emerging cellular events with therapy. Palbociclib and venetoclax induce delta-like layering and formation of separate cell clusters, respectively. The functionally broad targeted anticancer agents induced diverse compositions of tumor microenvironmental states (legend). Cell graphs with feature values and physical cellular interactions based on proximity serve as input for unsupervised clustering methods to find the microanatomy of the tumor tissue with a drug response. **b** *Left* Systematic testing of unsupervised discovery of tissue architecture with graphs (UTAG)^1^ with different maximum Euclidean distances for graph construction (y-axis) and different clustering resolutions (x-axis; top heatmap). Average proportion of UTAG clusters within formerly defined supervised maps^3^; bottom heatmap. *Right* Uniform Manifold Approximation and Projection embedding (UMAP) with UTAG identified clusters (7.5μm max distance; resolution = 0.3, *n* = 6598 cells) and positional plot colored by cellular phenotype in local panobinostat drug response after three days of exposure in MMTV-PyMT tumors. Treatment-specific clusters are marked by pink dotted lines. Galectin-3 phenotypes propagating across clusters in UMAP are depicted by arrows; magnified view. **c** *Left* Spatial organization and abundance changes in the TME of the assay vs control region as measured by GraphCompass. Top to bottom: panels visualize the number of cells, graph density, and spatial topology differences using the portrait method, where dot size reflects consistency across *n* = 3 biological replicates. Larger, darker dots indicate low variance and high confidence over samples between assay and control conditions. Significant *p*-values are shown. *Right* partition-based graph abstraction (PAGA) estimating the connectivity of manifold partitions through galectin-3 and galectin-3 linking functional phenotypes across space. Legend as in b. **d** Spatial organization of the microanatomical domains and statistical testing as identified by UTAG and GraphCompass (n = 3 biological replicates) of palbociclib (*top*) and venetoclax (*bottom*) treatment for three days. Significant *p*-values are shown. See also Repository Supplement S6 and S7; 10.6084/m9.figshare.32513355. The [0,0] coordinate is the drug release site in the spatial graphs; the direction of the drug release is upward. c., cells; t. tumor; AP, antigen presenting; CSC, cancer stem cells; gal-3, galectin-3**;** M, macrophages

Mouse mammary tumors were implanted with panobinostat-loaded microdevices. After three days of drug exposure, we performed analysis with unsupervised discovery of tissue architecture with graphs (UTAG^1^)—a spatial clustering method that effectively identifies anatomical domains in normal tissues. We implemented a systematic approach using varying levels of maximum Euclidean distances as well as clustering resolutions and quantitatively compared the newly identified macroscopic domains with maps identified previously using supervised cell classification (Fig. 1b, left, Supplementary Materials and Repository Supplement S1-3; 10.6084/m9.figshare.32513355). Our results suggest that finding treatment-specific and biologically interpretable anatomical domains inside the tumor bed can be achieved with distance-based clustering methods using 7.5–12.5 μm distances with a resolution of 0.2–0.3 if unsupervised learning methods are integrated in the computational search (Fig. 1b, left).

Applying the UTAG algorithm with the highest score parameter (7.5 μm distance; 0.3 resolution) in MIMA, we identified a treatment-specific antigen-presenting myeloid cell (APC) cluster localized immediately proximal to the drug site (cl. 1) followed by an immunogenic cell death (ICD) cluster (cl. 0) and two galectin-3 clusters (cl. 2 and 3). The mixed galectin-3 myeloid cell and cancer stem cell cluster (cl. 3) propagated into the other treatment-specific clusters in the uniform manifold approximation and projection (Fig. 1b, right). Using GraphCompass,^2^ which encompasses a statistical analysis framework, we found the ICD cluster (*p* = 0.015) and the galectin-3 mixed cl. 3 (*p* = 0.031) to be significantly enriched in treatment-specific conditions (Fig. 1c, left). Additionally, partition-based graph abstraction^4^ estimating the connectivity of manifold partitions implied galectin-3 to be linked to mesenchymal cells (cl. 12) through an immune suppressive subset (cl. 10; Fig. 1c, right). This spatial co-occurrence of galectin-3 with other clusters was confirmed by cell‒cell interaction analysis and neighborhood enrichment assessment, which also revealed ICD and protumor macrophages to be enriched with APCs and proliferating tumor, respectively (Repository Supplement S4; 10.6084/m9.figshare.32513355). Last, while clustering using a Leiden algorithm did not identify antigen-presenting neutrophils as shown before,^3^ UTAG detected this panobinostat-induced cellular phenotype (Repository Supplement S1 and S4; 10.6084/m9.figshare.32513355). Thus, implementing unsupervised architecture methods, including spatial information versus sole expression analysis in MIMA, provides superiority both in terms of (i) capturing rare events as well as (ii) finding cellular phenotypes that are likely functionally interconnected with therapy.

We further investigated panobinostat efficacy at the extended (day eight) time point. We no longer observed APCs, suggesting a loss of the antitumorigenic phenotypes. Instead, we found a large proximal assay area to be dominated by therapy resistance, specifically, dying and immune-suppressive myeloid cells (cl. 2), followed by cancer stem cells (cl. 6), mesenchymal cells (cl.14), and protumorigenic macrophages (cl. 10; Repository Supplement S5; 10.6084/m9.figshare.32513355). Along with these resistance domains, UTAG also discovered a novel cell phenotype of sparse, individual, cytotoxic B cells expressing CD45R, Granzyme B, calreticulin, and galectin-3 (cl. 13), whose functional role warrants further investigation. Overall, our findings suggest galectin-3 as (i) having a pleiotropic function and (ii) being a central phenotype linking the panobinostat treatment-specific cellular events across space (Fig. 1b) as well as across time (Repository Supplement S5; 10.6084/m9.figshare.32513355).

By analyzing macroscopic domains induced in MIMA, we found different targeted anticancer agents induced enrichment of a broad range of immune and nonimmune stromal cells organized into specific spatial patterns, some of which resembled water‒land interactions in geology^5^ (Fig. 1a, right). Panobinostat cell phenotypes formed bay-like layers with increasing distance from the well, with some antitumorigenic immune cells disappearing and protumorigenic resistant cells appearing at a later time (Fig. 1a, right). This result provided the first evidence of dynamic TME architecture assessment with drug treatment in MIMA. Palbociclib and venetoclax induced significant recruitment of distinct myeloid subsets, in association with endothelial cells and pericytes that formed a delta-like structure or appeared as split clusters, respectively (Fig. 1a, right and 1d). This suggests panobinostat and palbociclib layer phenotypes to be more likely functionally interconnected in cause-consequence association; while venetoclax-induced spatial clusters might have formed as independent domains.

## Conclusion

We formerly showed that MIMA-predicted drug combinations were highly effective when administered systemically. This approach was demonstrated in mouse models of breast cancer but is already being adapted for human cancers. Here, the presented systematic analysis of spatially resolved treatment responses allows (i) establishment of parameters for reproducible identification of anatomical domains, providing superiority in terms of identification of (ii) novel cell types (cytotoxic B cells), (iii) rare cells (antigen-presenting neutrophils), and (iv) functional phenotypes linking treatment-specific events across space and time (galectin-3). We show functionally broad targeted anticancer agents to generate distinct spatial patterns reminiscent of water‒land interactions,^5^ allowing (v) dynamic systems assessment and identification of likely cause-consequence cellular associations. We foresee the novel integration and the acquired results to be readily applicable to a broad range of preclinical and clinical studies involving MIMA as well as systemic treatments, longitudinal studies and mathematical modeling.

## Material availability

This study did not generate new materials.

## Supplementary information

Supplementary Information

## Data Availability

The source raw registered images for feature extraction are provided as a collection of images at the figshare.com repository 10.6084/m9.figshare.19719421, 10.6084/m9.figshare.19719499, 10.6084/m9.figshare.19719514. The image analysis pipeline using publicly available functions and the associated scripts that support the findings of this study are available within the article, in the Supplementary Materials and Information (track ID: 10.6084/m9.figshare.32513355), or from the corresponding authors upon reasonable request.
