# Supplementary material for: Discovery of dynamic tumor microenvironment architecture with targeted agents in the multiplex implantable microdevice assay
Source: Signal Transduct Target Ther. 2026 Jul 10;11:270. doi: 10.1038/s41392-026-02824-z (PMC13350716; doi:10.1038/s41392-026-02824-z)
Supplement: Supplementary file 1 — Supplementary Information [file 41392_2026_2824_MOESM1_ESM.docx]

Supplementary Materials for

Discovery of Dynamic Tumor Microenvironment Architecture with Targeted Agents in the Multiplex Implantable Microdevice Assay

Authors: Juraj Jakubik,^1,2,3^ Zuzana Tatarova^1,2,3,4*^* Correspondence to: [zuzana.tatarova@dkfz-heidelberg.de](mailto:zuzana.tatarova@dkfz-heidelberg.de)

**This PDF file includes:**

Materials and Methods

**Microdevice implantation studies and sample collection**

Multiplex implantable microdevice assay (MIMA) guides the identification and validation of synergistic treatment combinations of targeted anti-cancer agents with immune-based therapies including immune checkpoint blockade, anti-CSF1R and anti-CD40.^1^ Details of the MIMA system and of the sample collection were described previously (^1^). Briefly, mouse mammary tumor virus-polyoma middle tumor-antigen tumor bearing mice^2^ were implanted with a cylindrical microdevices (5.5mm in length)^3^ packed with drugs mixed with Polyethylene glycol (MW 1450, Polysciences) polymer at the concentrations 25%, 25%, 30% for panobinostat, palbociclib and venetoclax, respectively. After three and eight days of drug exposure, the tumors were explanted, and the derived formalin-fixed paraffin-embedded sections were stained at the drug/tumor cross-section with multiplex immunohistochemistry^1^ using 22 biomarkers. See also Supplementary Figure 1 (Fig. S1) of the online Repository Supplement; track ID: https . The iteratively digitized images were co-registered using MATLAB (The MathWorks, Inc., Natic, MA, version 2019b) utilizing the detectSURFFeatures algorithm. To quantify single cell signal mean intensity as defined by mask, the signal was scaled to a range 0-1. The experimental region of interest (ROI) of the panobinostat assay area was defined directly above the reservoir with size of 750μm x 1375μm (4190 cells), and was compared to the rest of the tissue section with dimensions 1500μm x 1625μm (2408 cells). Quality of the single cell data was ensured by excluding deformed (folded), lost or unevenly stained tissue (border effects). All animal studies were conducted in accordance with protocols approved by IACUC at OHSU (protocol number: IP00000956).

**Unsupervised Leiden algorithm run and UTAG selection using MIMA treatment data**

Scaled raw marker expression and the centroid coordinate data for each segmented cell was processed using Scanpy^4^ (v1.11.5). Each marker was validated for specificity. Positive control tissues^1^ were used to help to define the single parameter threshold for positivity by manual gating. We have conducted a head-to-head comparison of seven clustering approaches applied to the same TME dataset (6598 cells x 22 markers; Leiden^5^, Louvain^6^, K-means^7^, Hierarchical^8^, DBSCAN^9^, and Squidpy^10^, UTAG^11^). Here we used default parameters (and a random state of 0) provided in the scripts as a starting point. UTAG’s primary advantage was identification of biologically meaningful spatial microdomains resembling those identified by supervised cell classification in our former study.^1^ Fig. S2 contains a table comparing features of the clustering methods as well as the visual benchmarking of the spatial domain identification. All plots use the Tab20 color palette for consistent visual comparison. Unsupervised discovery of tissue architecture with graphs (UTAG^11^) was implemented within the Python package named utag (v0.1.0). This involved dimensionality reduction using UMAP with a neighborhood size of n_neighbors=75 and unsupervised clustering with Leiden algorithm^5^ (Fig. S1), employing resolution=0.4 (from 0.3, 0.4 and 0.5 tested). To assess the distribution of macroscopic structural domains, we systematically applied different maximum distances and clustering resolutions (Fig. S3a) which were ranging between 7.5-37.5μm and 0.025-0.4 values, respectively. Systematic quantitative evaluation to search for ideal parameters for TME architecture discovery with therapy response were performed using the panobinostat day 3 assay area of the MIMA^1^ data (750μm x 1375μm ROI with 4190 cells). Specifically, first we generated maps of known major phenotypes^1^ with panobinostat response: antigen presenting cells, immunogenic cell death, cancer stem cells and galectin-3 with increasing distance from the well. Blurring the 8-bit scatter plot image using gaussian blur, followed by automatic threshold application and edge detection was used to define the phenotype maps (Fig. S3b). Area % was quantified for each UTAG cluster within the matched zone map. If no cluster was present within the map, the area % was set to 0% and the average of four zones was then used to generate the heatmap (in Fig. 1b, left). Distance-based clustering methods using 7.5-12.5μm maximum distances with resolution 0.2-0.3 detect biologically interpretable tumor microenvironment anatomical domains. Large distances (37.5μm) restricted to low resolutions (0.025) will only detect the tumor/stroma interface (Fig. S3a bottom left). Systematic MIMA/UTAG testing inside the tumor suggests that medium to high distances (20-37.5μm) with 0.1-0.3 resolution should be avoided due to likely artifact detection (Fig. S3a right). Generally, the lower the maximum distance, the higher the resolution that should be implemented to reliably find treatment induced TME changes.

**Data Preprocessing and Joint Clustering**

To compare spatial organization in assay ROI with control tumor tissue (n=3 each), we merged all our datasets into a single anndata object. Cell markers CD3 and FOXP3 were removed by staining quality (described in staining quality section). Joint Leiden clustering was performed on the concatenated dataset. Dimensionality reduction was first performed using PCA (scanpy.pp.pca), followed by k-nearest-neighbors graph construction (scanpy.pp.neighbors), and Leiden community detection (scanpy.tl.leiden) at a resolution of 0.4 (panobinostat) and 0.3 (palbociclib, venetoclax). All three steps used random_state=42 to ensure fully deterministic and reproducible cluster assignments across independent runs. These clusters were then labeled according to our expert-defined cell types (Fig. S4a, b). Additionally, cell types represented by fewer than 5 cells in at least 4 of the 6 samples were excluded from visualization to ensure sufficient statistical sampling.

**Spatial Graph Analysis (GraphCompass)**

The GraphCompass^12^ (v0.2.5) graph analysis framework was used to quantify the spatial distribution of each cell type between samples (Fig. 1c, left). We computed Portrait Divergence (distance) values between each pair of samples using the portrait method, which compares the distribution of shortest path lengths between each graph. Difference in spatial topology (e.g., dispersion vs. aggregated point patterns) was measured apart from cell density.

**Spatial analysis of cell-cell communications using Squidpy**^10^

Pre-processed dataset containing 6,598 cells and 22 protein markers was analyzed. Cells were clustered into 13 distinct cell type populations using UTAG^11^ and involved spatial coordinates (X and Y centroids) for each cell. Spatial analysis was performed using Scanpy and Squidpy (v1.7.0) using NumPy^13^ (v2.3.5) amd Pandas^14^ (v2.3.3) in Python using all default parameters for each method unless explicitly stated otherwise. A spatial neighborhood graph was constructed using Delaunay triangulation (sq.gr.spatial_neighbors(), coord_type='generic', delaunay=True, percentile=95), This method finds the neighbors based on actual spatial proximity rather than fixed k-nearest neighbors, resulting in an average of 5.6 neighbors per cell. The resulting connectivity matrix was stored in adata.obsp['spatial_connectivities']. Neighborhood enrichment analysis of spatial co-localization patterns was assessed using permutation testing (sq.gr.nhood_enrichment()) with 1,000 permutations (n_perms=1000, seed=42) in Fig. S4g. Additionally, we performed spatial interaction analysis with k-nearest neighbors (KNN^15^). Spatial graph was constructed using sklearn.neighbors.kneighbors_graph() with k=6 neighbors based on Euclidean distance between cell centroids. Cell-cell interaction matrices were computed from spatial graph using NetworkX^16^. Following three complementary visualizations were generated (Fig. S4d-f): chord diagram with arc segments, network graph with edge widths and the heatmap showing frequent/full interactions probability matrix with other clusters from UTAG analysis.

A total of 22, 27, 12, 15 markers were selected for panobinostat day 3, panobinostat day 8 (Fig. S5), palbociclib day 3 and venetoclax day 3 (Fig. 1d and S6, S7), respectively for downstream analysis. Macroscopically, panobinostat-induced tumor microenvironmental domains formed bay-like layers with disappearing and emerging phenotypes over time. Palbociclib and venetoclax associated domains were arranged as tree/delta-like layers and split clusters, respectively, with layering being suggestive of cellular communication. Additionally, the delta-like structure is suggestive of fast single cell interactions; while the bay-like phenotype might reflect slow cell communication^17^ resulting in response and/or resistance. Panobinostat, venetoclax and palbociclib day 3 conditions used n=3 replicates from 2-3 tumors from 2-3 mice. The integrated MIMA/UTAG tool enables identification of anatomical domains independent of DAPI (nucleus) involvement. DAPI was involved in the palbociclib implants; while it was left out from analysis for panobinostat and venetoclax conditions. Probe combination, number of cells analyzed within number of clusters are in the legends.

**REFERENCES**

1. Tatarova, Z. et al. A multiplex implantable microdevice assay identifies synergistic combinations of cancer immunotherapies and conventional drugs. *Nat. Biotechnol.* **40**, 1823–1833 (2022).

2. Guy, C. T., Cardiff, R. D. & Muller, W. J. Induction of mammary tumors by expression of polyomavirus middle T oncogene: a transgenic mouse model for metastatic disease. *Mol. Cell. Biol.* **12**, 954–961 (1992).

3. Jonas, O. et al. An implantable microdevice to perform high-throughput in vivo drug sensitivity testing in tumors. *Sci. Transl. Med.* **7**, 284ra57 (2015).

4. Wolf, F. A., Angerer, P. & Theis, F. J. SCANPY: large-scale single-cell gene expression data analysis. *Genome Biol.* **19**, 15 (2018).

5. Traag, V. A., Waltman, L. & van Eck, N. J. From Louvain to Leiden: guaranteeing well-connected communities. *Sci. Rep.* **9**, 5233 (2019).

6. Blondel, V. D., Guillaume, J.-L., Lambiotte, R. & Lefebvre, E. Fast Unfolding of Communities in Large Networks. *Journal of Statistical Mechanics Theory and Experiment* **2008**, (2008).

7. Ikotun, A. M., Ezugwu, A. E., Abualigah, L., Abuhaija, B. & Heming, J. K-means clustering algorithms: A comprehensive review, variants analysis, and advances in the era of big data. *Inf. Sci. (Ny)* **622**, 178–210 (2023).

8. Cohen-Addad, V., Kanade, V., Mallmann-Trenn, F. & Mathieu, C. Hierarchical clustering: Objective functions and algorithms. in *Proceedings of the Twenty-Ninth Annual ACM-SIAM Symposium on Discrete Algorithms* 378–397 (Society for Industrial and Applied Mathematics, Philadelphia, PA, 2018). doi:10.1137/1.9781611975031.26.

9. Ester, M., Kriegel, H., Sander, J. & Xu, X. A density-based algorithm for discovering clusters in large spatial databases with noise. *KDD* 226–231 (1996) doi:10.5555/3001460.3001507.

10. Palla, G. et al. Squidpy: a scalable framework for spatial omics analysis. *Nat. Methods* **19**, 171–178 (2022).

11. Kim, J. et al. Unsupervised discovery of tissue architecture in multiplexed imaging. *Nat. Methods* **19**, 1653–1661 (2022).

12. Ali, M. et al. GraphCompass: spatial metrics for differential analyses of cell organization across conditions. *Bioinformatics* **40**, i548–i557 (2024).

13. Harris, C. R. et al. Array programming with NumPy. *Nature* **585**, 357–362 (2020).

14. McKinney, W. Data Structures for Statistical Computing in Python. in *Proceedings of the Python in Science Conference* 56–61 (SciPy, 2010). doi:10.25080/majora-92bf1922-00a.

15. Goldberger, J., Roweis, S., Hinton, G. E. & Salakhutdinov, R. Neighbourhood Components Analysis. *Neural Inf Process Syst* 513–520 (2004).

16. Proceedings of the Python in Science Conference (SciPy): Exploring Network Structure, Dynamics, and Function using NetworkX. http://conference.scipy.org.s3-website-us-east-1.amazonaws.com/proceedings/scipy2008/paper_2/.

17. Charles C. Bates (2). Rational theory of delta formation. *Am. Assoc. Pet. Geol. Bull.* **37**, 2119–2162 (1953).
